# Technology-Based Interventions to Reduce Sugar-Sweetened Beverages among Adolescents: A Scoping Review

**DOI:** 10.3390/ijerph20237101

**Published:** 2023-11-23

**Authors:** Chidinma Ezike, Keith Da Silva

**Affiliations:** 1 School of Public Health, University of Saskatchewan, Saskatoon, SK S7N 5E5, Canada; 2 College of Dentistry, University of Saskatchewan, Saskatoon, SK S7N 5E5, Canada; keith.dasilva@usask.ca

**Keywords:** interventions, sugar-sweetened beverages, technology, adolescents

## Abstract

This scoping review investigates the effectiveness of technology-based interventions in reducing sugar-sweetened beverage (SSB) consumption among adolescents. The rise in SSB consumption among young individuals has become a global public health concern due to its association with obesity, diabetes, and various other health problems. The purpose of this scoping review is to map out and examine the various technology-based interventions used in reducing sugar-sweetened beverages among children and adolescents. A systematic search of three databases using the PRISMA guideline was followed, and 474 articles were retrieved. Seven articles met the inclusion criteria and the critical appraisal using the critical appraisal skill program (CASP). The seven articles underwent both descriptive and thematic analysis. Four technology-based interventions were identified from the selected articles, which include smartphone apps, online or web-based tools, text messages, and social marketing strategies. Our findings suggest that these interventions hold promise in improving adolescents’ eating patterns and health outcomes associated with SSB intake, highlighting their potential as useful strategies in resolving this urgent public health concern.

## 1. Introduction

The term “sugar-sweetened beverages” (SSBs) refers to liquids that have added sugar in a variety of ways, such as regular sodas, fruit drinks, energy drinks, sweetened coffees and teas, and sweetened dairy drinks [1]. Sugar-sweetened beverages (SSBs) are a significant source of free sugars in the diet [2,3]. In the past few years, the intake of SSBs has rapidly expanded among adolescents and children, becoming a preferred beverage for millions of people globally [2,4]. The World Health Organization (WHO) recommends limiting free sugar intake in adults and kids to less than 10% of total calorie intake [5]. Unfortunately, Canadians aged one year and above ingest 110 g of total sugars, which is 20% of their total calorie intake [6].

The consumption of these beverages is a major public health problem as their excessive intake has been linked to obesity, type II diabetes, hypertension, metabolic syndrome, and dental caries [2,7,8]. A study from the Harvard School of Public Health reported that SSB consumption has caused roughly 180,000 obesity-related deaths, 133,000 deaths from diabetes, 44,000 cardiovascular disease deaths, and 6000 cancer-related deaths [9]. The researchers discovered that low- and middle-income nations accounted for 78% of these deaths [9]. In Canada, dietary risks (e.g., diets low in whole grains, fruits, and vegetables; high in red and processed meat; and sugar-sweetened beverages) are one of the top three risk factors for worldwide accountable deaths [10].

Several approaches have been made to address this issue, primarily through interventions that focus on environmental and behavioral modifications. Commonly used interventions include health literacy that teaches parents, students, teachers, and other caregivers the effects of excessive consumption of SSBs, the substitution of SSBs with plain and portable water, improved nutritional labeling of non-alcoholic beverages, enacting policies in schools and workplaces to limit the availability of SSBs, increased taxation of SSB products, counseling, social marketing, technology-based intervention, and so on [1,11,12]. Technology-based interventions have been created as strategies to combat excessive SSB consumption among adolescents since it has become such an important component of their daily lives. Examples of commonly used technology-based interventions include social networking sites, smartphone apps, online weight-control tools, and active video games [13].

Children and adolescents increasingly use smartphones, tablets, and other mobile devices for learning and entertainment [14]. Adolescents are extremely proficient with technology, with over 93% using the Internet regularly [15]. Due to the high number of teenagers’ use of technologies, there is potential to promote health using new media platforms that are essential to young culture. Technology-based interventions have been used successfully to prevent obesity, increase physical activity, improve diet and eating habits, smoking cessation, and alcohol reduction in adults and teenagers [13,16,17]. There have been research and reviews on the effect of technology-based approaches on weight management, physical activity, and sedentary life, but few reviews have centered on its effect on sugar-sweetened beverage reduction, and no review has centered on the effects of technology-based intervention on SSB reduction among adolescents. The purpose of this scoping review is to map out various technology-based strategies and give a summary of them.

## 2. Materials and Methods

The scoping review was carried out between May and July 2023. This scoping review was not registered. The review was guided by the following question: How effective is technology-based intervention in reducing sugar-sweetened beverages among children and adolescents? The importance of this question is to highlight important public health strategies for this population in adopting a healthy diet.

Search Strategy and Selection Criteria: To find relevant terms to include in the search strategy, the PICO framework, which stands for population/intervention/comparison and outcome, was first used. A comprehensive search of peer-reviewed publications was conducted with the help of a university-based librarian using MeSH words and keywords in Medline Ovid, PUBMED, and CINAHL databases. We finally performed citation searching to ensure a maximum result. Selected articles were exported to the Zotero referencing manager and also as a CSV file from which duplicates were removed. An example of the search strategy designed in Medline Ovid is seen in Supplementary File S1 (in Supplementary Materials).

Using the Preferred Reported Items in Systematic Reviews and Meta-Analysis (PRISMA) flowchart [18], the screening and selection process was performed. Eligibility was verified by two reviewers based on title and abstract screening for inclusion criteria, which was then followed by retrieving and evaluating the full-text articles.

Inclusion Criteria: Articles whose participants and intervention are directly delivered to adolescents (8–19 years); technology-based interventions; school-based and community-based intervention; articles in the English language and within 10 years of publication; articles whose study design are randomized control trial (RCT) and clustered RCT; and articles with an empirical data report.

Exclusion Criteria: articles whose participants are adults or all age groups; non-technology-based interventions; family-based interventions, articles in a language older than English and above 10 years publication date; systematic reviews articles; and quasi-experimental study design.

Quality Appraisal: We used the Critical Appraisal Skills Program (CASP) tool (for RCT) to perform a critical appraisal of the selected articles. This tool (consisting of 11 questions) assesses the quality of the articles.

Data Extraction and Analysis: Studies that met the inclusion and appraisal criteria were summarized and presented in tables using the following characteristics: study references (author, year of publication, trial design, and place of study), study duration, type of intervention, (frequency, duration, and intensity of activities), duration of intervention and follow-up, participants’ characteristics (mean age, sex, and other socio-demographic features available), outcome measured, and overall findings [19]. The extracted information was then used to make a descriptive analysis of the study and thematically analyzed.

## 3. Results

**Figure 1:**
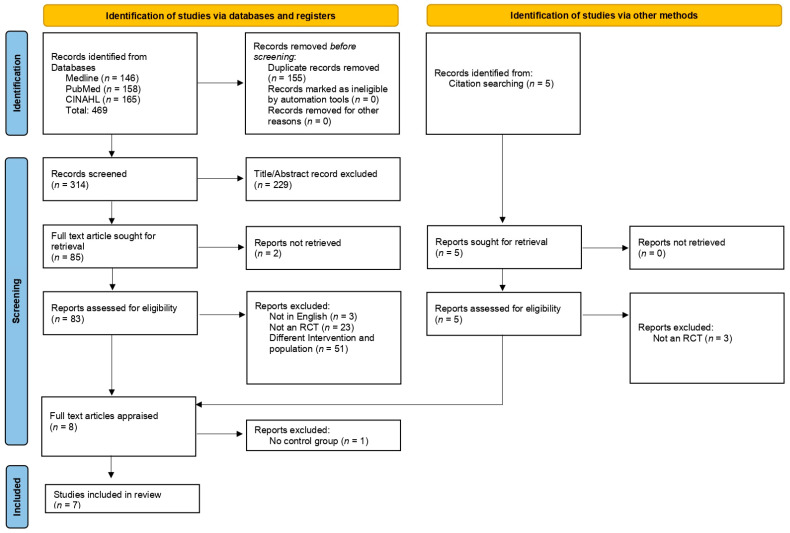
PRISMA flowchart of study selection.

A summary of the search process is shown in the PRISMA flow diagram (Figure 1). A total of 474 articles were identified, with 7 articles extracted for the review.

### 3.1. Descriptive Analysis

Of the seven articles used for this Scopus review, six (85.71%) articles used mixed methods except for one article [20], which used only quantitative methods. The majority of the studies were from developed countries like the United States, Australia, the Netherlands, and Spain. Only one article was from a developing country (Brazil). Across the seven trials, adolescents between 9 and 18 years took part. Studies used were predominantly cluster randomized control trials (*n* = 4, 57.14%) [20,21,22,23] and randomized control trials (*n* = 3, 42.85%) [24,25,26].

### 3.2. Quality Appraisal

The CASP tool for RCT was used to appraise the articles (Table 1). Eight articles were appraised, of which seven articles met the criteria. The article [27] did not meet the criteria because it did not use a randomized method, no control group, and no confidence interval was reported. The seven studies all met the following CASP requirements: specified study population and participant age range, as well as study goal or aim. Of the selected articles, only four articles [21,23,24,26] generated a confidence interval around the outcomes.

### 3.3. Interventions Characteristics

Table 2 shows the summary of the articles. Three articles used smartphone app interventions [23,25,26], one article used internet tools [2] to deliver the intervention, one article used text messages [24], and two last articles used the social marketing strategy [20,22]. Four studies cited a theoretical basis for their intervention, which are cognitive behavioral theory [21], self-determination theory [22,23], and social cognitive theory [23,26].

### 3.4. Thematic Analysis

#### 3.4.1. Smartphone App Intervention

In this present review, three studies [23,25,26] were used to successfully reduce SSB consumption. In the study by Smith et al. (2014), a smartphone app was created to support the delivery of the expanded interactive sessions by giving users a way to keep track of their actions, set objectives, and evaluate their competency in using real-time technology. The app additionally offered personalized educational and encouraging messages via “push prompts”. Measurements include anthropometric values, physical activity, SSB consumption, screen time, and RT competency. Results showed significant improvements in screen time, SSB consumption, and RT skills. No effects were seen in BMI and physical activity [23].

Nollen et al., 2014 conducted a 12-week mobile technology (MT) intervention for girls aged 9–14 who are members of the afterschool program using a MyPal A626 handheld computer. The intervention included modules that targeted fruits and vegetables (FVs), sugar-sweetened beverages, and screen time. The MT intervention encouraged goal-setting and self-monitoring in real time while providing advice, feedback, and encouragement for the target behaviors. The mobile app also included a song-based reward system (reinforcement) that gave participants one song per day if they answered 80% of daily prompts. Measures include assessing device utilization, intake of FVs, SSBs, screen time, and BMI. Results showed that the intervention group showed increased FV consumption and decreased SSB consumption. No difference was seen in screen time and BMI between the groups [25].

The third study by Chen et al., 2019, assessed the short-term impact of smartphone-based intervention in overweight Chinese-American adolescents. The intervention was carried out in the USA for 12 months using a Fitbit Flex app. The Fitbit Flex app and the online program were utilized to monitor physical activity, sedentary activity, and nutritional growth (by tracking consumption of FVs, SSBs, and water per day); set personal goals; track advances towards achieving goals; offer advice for daily activities; and offer tips on sustaining a healthy weight. Measures include anthropometry, physical–sedentary activity, sedentary activity, food consumption, and Pediatric Quality of Life (PQOL)-Adolescents. The culturally appropriate smartphone-based intervention was capable of reducing SSB, fast-food consumption, and sedentary activity, which in general reduces obesity and improves a healthy lifestyle [26].

#### 3.4.2. Internet or Web-Based Intervention

Brito et al., 2019, organized a web-based intervention (StayFit) to encourage and guide weight control and healthy eating habits. The intervention was carried out in Brazil for 12 months, and it involved 16 sessions which were carried out through a virtual learning environment. Participants were encouraged to read the materials and complete the tasks, logs, and feedback from the various sessions. All materials were culturally adapted to suit the language, culture, and context of Brazil. Measurements collected at baseline and end of intervention include food consumption, anthropometry, physical activity level, and sedentary behavior. Results revealed a 43% increased chance of consuming beans and a 35% chance of reducing SSB intake. No differences were recorded in anthropometric parameters [21].

#### 3.4.3. Text Messages Intervention

In this present review, Gustafson et al., 2019 conducted an 8-week, mentor-led text-messaging intervention among rural adolescents in Kentucky and North Carolina aimed at promoting fruits, vegetables, and healthy beverage intake. Nutrition undergraduate students were trained to mentor high school students through interaction with text messages. The first week was termed a “warm-up week”, in which participants and students worked out any communication issues and came to know their mentor. It was a two-way communication as messages were sent forth and back between the undergraduates and participants. The primary outcome measured was fruit and vegetable intake. The secondary outcomes measured were sugar-sweetened beverage intake, body mass index (BMI) Z-score, home food availability, purchasing habits, self-efficacy, and goal setting related to healthy eating. There was a statistically significant impact on the intake of fruit and vegetable servings/day and a high likelihood of adopting goals for healthy eating habits [24].

#### 3.4.4. Social Marketing Intervention

Two articles [20,22] from our present review used social marketing intervention to reduce SSB consumption among adolescents. In one of the studies, Tarro et al., 2019, carried out a 10-month parallel-cluster randomized controlled study aimed at promoting a healthy lifestyle of younger peers by developing and implementing peer-led social marketing health activities (EYTO-Kids) in a disadvantaged area in Reus (Spain). The sample size used was eight primary schools (*n* = 375, 9–11 years) and nine high school students acting as the adolescent creators (*n* = 94 ACs, 12–14 years). The EYTO-Kids project uses social marketing to promote health among peers; the intervention was in five stages; ACs were trained in nutrition, social marketing, and communication skills; AC designed the activities standardized by writing the theatre script. Additionally, it employs a variety of techniques, including funny games, visual aids, and teen food product tasting. Measurements included the proportion of children who ate more than one portion of fruit per day, who did moderate to strenuous physical exercise, who ate ≥1 vegetable daily, and SSB consumption every day. EYTO peer-led project effectively increased physical activities decreased SSBs and fast-food consumption, and screen time [20].

Smit et al. (2020) [22] conducted a three-arm cluster randomized control study with schools, dividing them into three groups at random: the SNI group, the active control group, and the control group. Students in the SNI were exposed to classmates from their classrooms who had been chosen and educated as influencers to encourage water drinking as an alternative to SSB intake. The active control group simultaneously exposed all the kids to a presentation on the advantages of drinking water. It was based on the ideas of mass media campaigns. The students in the control group did not receive any intervention. It was a 5-month study from February to June 2018. The participants consisted of 451 children between 9 and 14 years old from 11 schools. Measures include peer nomination, water consumption, SSB consumption, descriptive norms, and injunctive norms. The study showed that the SNI group consumed less SSB per day compared to those in the active control and control conditions. No differences were found between conditions for water consumption [22].

## 4. Discussion

This scoping review aimed to map out and summarize technology-based interventions in reducing adolescent sugar-sweetened beverage (SSB) consumption. It identified seven studies focusing on technology-based strategies to decrease SSB intake among adolescents in primary and high schools. Despite the growing popularity of employing technology for promoting healthier behaviors among young populations, there remains a notable gap in our understanding of the effectiveness of these methods, specifically for adolescents. Globally, the adoption of digital tools for health improvement is increasing, especially among young people who are exposed to the Internet from an early age [28]. Previous studies have demonstrated the effectiveness of digital interventions in promoting various health-related behaviors among adolescents, including positive body image [29], mental health [30], substance use prevention [31], and physical activity promotion [32]. The interventions in this review included smartphone apps [23,25,26], internet tools [21], text messages [24], and social marketing strategies [20,22].

Mobile apps, given their widespread use, convenience, and cost-effectiveness [33], are considered an ideal approach for effective interventions. They have been utilized for a range of purposes, including self-reporting [34], monitoring behavior change [35], tracking health status [36], data collection, feedback provision [35], and health education [34]. Enhancements in app entertainment and visualization features have been suggested to improve intervention efficiency [33]. One article in this review even incorporated a song-based reward system, offering participants a song per day for achieving an 80% response rate [25].

Web-based interventions, capitalizing on adolescents’ proclivity for digital interactions, offer benefits such as participant anonymity, reduced stigmatization concerns, cost-effectiveness, and high accessibility [37]. One previous systematic review by de Sousa et al. (2022) evaluated the effectiveness of web-based intervention in health behavioral change among adolescents. They found evidence that web-based interventions were successful in encouraging teenagers to improve their health-related behavior [38]. However, larger randomized controlled trials with representative samples are needed to further support these findings.

Short message service (SMS) or text messaging, known for its convenience, effectiveness, and ability to facilitate discreet communication [39], has shown success in promoting various healthy behavior changes among adolescents, including obesity management [40], diabetes management [41,42], mental health support [43], and reproductive health education [44,45]. The systematic review of text message health interventions suggests that SMS interventions have positive short-term behavioral outcomes.

The first systematic review of text message health interventions was conducted by Fjeldsoe et al., 2009. They reviewed 14 articles and reported that key components of SMS-delivered interventions included intervention initiation (by the researcher or participant), SMS dialogue initiation, SMS content customization (tailored or targeted), duration of intervention, research design, and engagement (one-way or interactive). They concluded that text messaging interventions have positive short-term behavioral outcomes [45]. In this present study, Gustafson et al., 2019, drew some key findings [24]. First, text messaging has the potential to be helpful in a variety of situations, including enhancing adolescents’ dietary consumption. Second, the results showed potential for future interventions aimed at enhancing adolescents’ shopping habits, a crucial target group for behavior change as teenagers grow into adults. Third, this approach made adolescents influencers of healthy food in their homes. Results show that the proportion of intervention participants reporting that “junk food” was available in their residences significantly decreased. The authors concluded that this intervention can be sustained in deprived rural areas due to the use of mentors and targeted text messages.

Social marketing, defined as “a social influence technology involving the design, implementation, and control of programs aimed at increasing the acceptability of a social idea or practice in one or more groups of target adopters” [46], has also been applied effectively in health interventions targeting diverse topics, including obesity [47], alcohol use [48], SSB reduction [49], mental health [50], and reproductive health [51]. Success in social marketing interventions has been attributed to several factors, including applying marketing criteria, financial support, and establishing social connections among program participants [52].

In summary, technology-based interventions offer several advantages in supporting adolescent health and behavior change, including real-time progress tracking, data collection capabilities, cost-effectiveness, and accessibility [53,54]. Tracking progress and health goals is very important in health interventions. Smartphone apps are used to monitor progress, track health behaviors, observe advancement, and provide motivation to reach health goals. Technology-based interventions also help in the personalization of intervention, according to the participants. In a study carried out by Liu et al. [55], participants mentioned that personalization in technology-based interventions according to their individual needs increased their sense of involvement and motivation for utilizing the intervention.

However, these interventions are not without limitations, such as the digital divide, varying tech literacy levels, privacy concerns, technical issues, and the challenge of long-term maintenance [54]. Therefore, a comprehensive approach that considers usability, accessibility, cultural sensitivity, privacy, and the integration of in-person support is essential to cater to the diverse needs and preferences of adolescents while mitigating potential drawbacks.

There are several important limitations to the study. First, only seven studies in total were considered in the scoping review. This limited dataset may not provide a comprehensive overview of the effectiveness of technology-based interventions in reducing sugar-sweetened beverage (SSB) consumption among adolescents. Second, the lack of exploration of cost-effectiveness in the included studies is a notable limitation. It is essential to comprehend the economic viability and cost-effectiveness of technology-based interventions to make well-informed decisions and carry out practical implementation in healthcare and public health contexts. In addition, the predominance of short intervention periods in the reviewed studies hinders the assessment of the long-term sustainability of behavior change brought about by these interventions. A focus on shorter timeframes limits our understanding of whether the observed effects persist over a more extended period, which is critical for assessing the interventions’ enduring impact. Hence, future studies should endeavor to include the cost-effectiveness of technology-based interventions and use a longer intervention timeline. A further systematic review quantifying the magnitude of the effect may also be warranted.

## 5. Conclusions

There has been an increase in sugar-sweetened beverages among adolescents, which can be seen in the high prevalence of chronic diseases among them. Hence, there is a clear need for an effective and innovative SSB reduction program. Adolescents are extremely proficient with technology, and due to the high use of technologies, there is potential to promote health using the new media platforms. Our findings suggest that technology-based interventions can be feasible to promote SSB consumption reduction among adolescents.

## Figures and Tables

**Table 1:** Critical appraisal of articles.

Author and Year	# CASP Criteria Met	# CASP Criteria Unmet	# Unclear Criteria	Proportion of Satisfied Criteria (%)	Assessment	Main Unmet Criteria
Nollen et al., 2014 [25]	8	2	1	8/11 (72.7%)	Good	No confidence interval was reported
Brito Beck da Silva et al., 2019 [21]	8	2	1	8/11 (72.7%)	Good	There were differences between the study groups at the baseline
Tarro et al., 2019 [20]	8	2	1	8/11 (72.7%)	Good	No confidence interval was reported
Smit et al., 2020 [22]	10	0	1	10/11 (90.9%)	Good	n/a
Sundborn et al., 2021 [27]	6	4	1	6/11 (54.5%)	Satisfactory	It did not use a randomized method. No control group. No confidence interval was reported.
Smith et al., 2014 [23]	10	1		10/11 (90.9%)	Good	No confidence interval was reported
Gustafson et al., 2019 [24]	10	0	1	10/11 (90.9%)	Good	n/a
Chen et al., 2019 [26]	10	0	1	10/11 (90.9%)	Good	n/a

n/a—Not Applicable.

**Table 2:** Summary Table of the Articles.

No	Reference	Country	Study Purpose	Study Design	Study Duration	Participant Characteristics	Theory	Intervention Type	Outcome Measured	Findings
1	Nollen et al., 2014 [25]	USA	To evaluate a mobile technology intervention to be implemented over a 12-week period and estimate effect sizes for a fully controlled trial.	RCT	From March 2011 to April 2012, participants were randomly assigned. In July 2012, the follow-up was completed.	Racial/ethnic minority girls aged 9–14 years	n/r	Mobile technology (MT) (12 weeks): fruits/vegetables (FV; weeks 1–4), sugar-sweetened beverages (weeks 5–8), and screen time (weeks 9–12); MT was delivered on a handheld computer; it helps the girls in goal setting, planning, and self-monitoring; There was feedback and reinforcement; MT was designed without gaming, social media, or text messaging formats.	Use of the MT was measured on each handheld computer at FV (baseline and week 4), SSB (baseline and week 8), and screening time (baseline and 12 weeks); Height and weight were also measured.	MT girls used the program on 63% of the days; they showed increased FVs and decreased SSBs consumption; screen time and BMI had no significant difference.
2	Brito Beck da Silva et al., 2019 [21]	Brazil	This study aimed to determine whether an adapted version of StayingFit (using the Internet) could help adolescents develop healthy eating habits and whether anthropometric measurements were accurate.	Clustered RCT	A 12-month community cluster trial was conducted from September 2016 to September 2017.	Male and female students in the 7th to 9th grades who attended 12 mid-sized public schools within Salvador’s public comprehensive education system	Cognitive-behavioral Therapy (CBT)	Stayfit Brazil is an online program that has 16 sessions. Reading the material and completing the activities, logs, and session feedback were all encouraged; it uses a generalist form of feedback; messages used are controlled by the Food Guide for the Brazilian Population and WHO recommendations for physical activity practice; adapted to meet the standards and values of Brazilian culture, language, and context.	At the start of the 12-month study and at the end, information on each student’s food consumption, anthropometry, degree of physical activity, and sedentary behavior were obtained. Data on demographics and socioeconomics were gathered at baseline.	The baseline data reported high rates of overweight, inactivity, and sedentary behavior. Students in the intervention group had a 43% higher likelihood of frequently eating beans and a 35% lower chance of regularly consuming SSBs at the completion of the follow-up period.
3	Tarro et al., 2019 [20]	Spain	The intervention engaged adolescent creators (ACs) to develop and implement peer-led and social marketing (SM) health-promoting activities in an effort to improve the healthy lifestyles of younger peers.	Clustered RCT	A 10-month intervention in which recruitment started in March 2016 and ended in December 2016	Children (9–11 years) from 20 primary schools and adolescents (12–14 years) from 8 high schools serving socioeconomically disadvantaged neighborhoods; the adolescents were used as adolescent creators (ACs)	n/r	EYTO-Kids project uses social marketing to promote health among peers; the intervention was in 5 stages; ACs were trained in nutrition, social marketing, and communication skills; AC designed the activities which were standardized by writing the theatre script. Additionally, it employs a variety of techniques, including funny games, visual aids, and teen food product tasting.	The proportion of school children who ate more than one portion of fruit per day, weighing about 150 g/portion; the proportion of children that engaged in ≥6 h of moderate to strenuous physical exercise per week as per international recommendations; the proportion of students eating ≥1 vegetable every day; percentage of SSBs consumption every day.	EYTO peer-led project was effective in increasing physical activities, decreasing SSBs and fast food consumption and also screen time.
4	Smit et al., 2021 [22]	Netherland	The aim is to evaluate the impact of a social network intervention (SNI) in enhancing children’s healthy drinking habits.	Three-arm cluster randomized controlled trial consisting of SNI group, active control group, and control group.	The study took place from February 2018 to June 2018	The participants consisted of 451 children between 9 and 14 years old from 11 schools.	Self-determination theory	Social network intervention (SNI) involves using selected and trained classmates as influencers to promote water consumption as an alternative to SSB consumption. The active control group was based on the mass media campaign principles.	Measures include peer nomination, water consumption, SSBs consumption, descriptive norms, and injunctive norms.	The study showed that the SNI group consumed less SSB per day compared to those in the active control and control conditions. No differences were found between conditions for water consumption.
5	Smith et al., 2014 [23]	Australia	The main objective of the current study was to assess the outcomes of ATLAS (Active Teen Leaders Avoiding Screen Time), a multi-component, school-based obesity prevention intervention utilizing smartphone technology.	Cluster RCT	A 20-week intervention that started from December 2012 to June 2013	361 adolescent boys (ages 12 to 14) from 14 high schools in New South Wales low-income neighborhoods who were thought to be at risk for obesity participated.	Self-determination theory and social cognitive theory	ATLAS is a multifaceted intervention that aims to prevent unhealthful weight gain by boosting physical activity, lowering screen time, and lowering SSB use. The smartphone app was created to support the delivery of improved school sports and interactive sessions by giving participants a way to keep track of their actions, establish objectives, and evaluate their proficiency with resistance training (RT) skills.	Measurements include anthropometric values, physical activity, SSB consumption, screen time, and RT competency.	There were significant improvements in screen time, SSB consumption, and RT skills. No effects were seen in BMI and physical activity.
6	Gustafson et al., 2019 [24]	USA	This study tested an eight-week mentor-led text-messaging intervention with rural adolescents aged 14 to 16 in order to increase their diet of fruits, vegetables, and healthy beverages.	RCT	An 8-week intervention study	530 Adolescents from 8 high schools in rural eastern Kentucky and rural eastern North Carolina	n/r	The “Go Big and Bring It Home” intervention is a mentor-led text messaging intervention where nutrition undergraduates were trained to mentor and send messages to participants through a mobile app. The majority of the SMS messages were emotive, and one of the challenges each week was to consume more fruits, vegetables, or low-calorie or healthy beverages. The “Group Me” mobile app was used by undergraduate nutrition students to send texts on Tuesdays and Saturdays of each week during the eight-week period.	The primary outcome measured was fruit and vegetable intake. The secondary outcomes measured were sugar-sweetened beverage intake, Body Mass Index (BMI) Z-score, home food availability, purchasing habits, self-efficacy, and goal setting related to healthy eating.	There was a statistically significant impact on the intake of fruit and vegetable servings/day There was no intervention effect on beverage intake.
7	Chen et al., 2019 [26]	USA	This study aimed to determine the short-term effectiveness of a smartphone-based intervention for overweight or obese Chinese-American adolescents and to investigate the factors linked to a lower body mass index (BMI).	RCT	The intervention lasted for 12 months.	Adolescents who are between 13–18 years old, self-identified as Chinese American, have BMI > 85th percentile, have the ability to carry out routine tasks, such as commuting to school, and own a smartphone and access to the Internet.	Social cognitive theory	The intervention had three main parts: respondents used a wearable sensor (Fitbit Flex) for six months, went through eight online educational modules for three months, and then got customized, biweekly text messages for three months after finishing the modules. The online tool and Fitbit Flex app were used to monitor changes in physical activity, sedentary behavior, and dietary intake. The educational module was culturally tailored to suit the concepts and beliefs of Chinese and Americans.	Measures employed in this study include anthropometry, physical-sedentary activity, sedentary activity, food consumption, and Pediatric Quality of Life (PQOL)-Adolescents.	The culturally appropriate smartphone-based intervention was capable of reducing SSB and fast food consumption, and sedentary activity, which in general reduces obesity and improves a healthy lifestyle.

n/r—Not reported.

## Data Availability

The data presented in this study are available upon request from the corresponding author.

## Supplementary Materials

The following supporting information can be downloaded at: https://www.mdpi.com/article/10.3390/ijerph20237101/s1, Supplementary File S1: Medline Ovid Search Strategy; Supplementary File S2: Preferred Reporting Items for Systematic reviews and Meta-Analyses extension for Scoping Reviews (PRISMA-ScR) Checklist [56].
